# Dynamics and factors influencing return migration to Sub-Saharan Africa: A systematic review

**DOI:** 10.1016/j.heliyon.2023.e18791

**Published:** 2023-07-28

**Authors:** Lemlem F. Weldemariam, Ayansina Ayanlade, Marion Borderon, Karoline Möslinger

**Affiliations:** a Department of Geography and Regional Research, University of Vienna, Universitätsstraße 7/5, 1010 Vienna, Austria; b Department of Rural Development and Agricultural Extension, Haramaya University, Dire Dawa, Ethiopia; c Department of Geography, Obafemi Awolowo University, Ile-Ife, Nigeria

**Keywords:** Return migration, Driving factors, Barriers, Sub-Saharan Africa

## Abstract

**Background:**

Return migration, the process of migrants returning to their countries of origin, is a vital aspect of migration that has received growing attention in recent years. One area of focus in the study of return migration is understanding the motivations that drive migrants to return home. Conducting a regional literature review on the dynamics and factors influencing return migration can provide valuable insights into this complex and dynamic phenomenon. It can inform policy development, help to address economic and social issues and contribute to our understanding of migration patterns and trends in the region.

**Purpose:**

This study, therefore, aims to understand the dynamics and factors that influence return migration to Sub-Saharan Africa, a region that has experienced significant outflows of migration over the past few decades. This study provides an understanding of the drivers of and barriers to return migration and how far they resonate with factors of mobility and immobility.

**Methods:**

A two-decade systematic literature review was conducted to determine the driving factors and barriers that influence return migration to Sub-Saharan Africa (SSA). Multivariate factors of return migration were examined based on the central question: why do migrants return to their homeland? The multiple-step systematic literature search covers a broad range of factors of return migration to sub-Saharan Africa.

**Result:**

The findings indicate complex scenarios influencing decisions to return to the region, with the interplay of driving factors as well as barriers to return. Social, personal, economic, and policy factors were among the major drivers of return migration, but social and personal drivers were found to be the major motivating factors of decisions to return to SSA, compared to policy and economic issues. The observed drivers and barriers to returning migration in SSA were categorized and discussed under thematic sections considering structural, individual and policy issues.

**Conclusion:**

The study concludes that migrants’ decisions to return could be determined by numerous structural factors, such as economic, political, social and environmental circumstances, both at the place of origin and at the destination. Therefore, the review could be a useful contribution to future research, governments, mobility-oriented organizations and policymakers for effective return-migration strategies.

## 1 Introduction

Global migration flow has grown significantly over the last decades. The statistical evidence from the World Migration Report in 2019 indicates 272 million migrants globally, which is approximately a four-fold increase since the 1960s, which accounts for about 3.5% of the global population [1]; the majority of them are from the Global South [2]. In 2019, more than 21 million sub-Saharan African migrants were living in a different African state from where they originated, and about 19 million were living on another continent [3]. This implies that migratory trajectories originating from this region may have considerable effects on countries of destination and origin. Migration, as an integrated global issue, and one that is intricately linked with social and economic development, has been discussed broadly in the literature [4].

In the context of studying the determinants of the migration decision process, Scholars have identified a range of factors that influence an individual's decision to migrate, including economic, social, political, and environmental factors. It has been highlighted that economic factors, such as the availability of jobs and income opportunities, have been found to be among the most significant determinants of migration in sub-Saharan Africa [[5], [6], [7], [8]]. Additionally, social factors such as family ties, social networks, and cultural values have also been found to play a crucial role in shaping migration decisions [9,10]. Political factors, including conflict and instability [[11], [12], [13]] as well as environmental factors such as drought and other natural disasters [9,14] have also been identified as factors in the decision to migrate. Most of the time, the migration-decision process is the result of a combination of economic, political, sociocultural, demographic and environmental drivers [15,16].

There are specific discussions in the literature on the drivers of return migration. Economic-linked reasons have been emphasized in return-migration studies [[17], [18], [19]]. For instance, using the economic downturn in Spain, Bastia (2011) reported how economic conditions in the destination determine migrants' decisions about returning to their country of origin during times of crisis. [19]; also find a causal positive effect of return migration on overall economic development as return migration leads to higher levels of development through improved income, labour, health, and educational outcomes. Some other studies pointed out that psychological and emotional factors are crucial in the decision to return [[20], [21], [22]]. Additionally [13], suggests that factors associated with social and relational motives such as family and living style contribute to migrants' return decisions. Media is a crucial factor in propagating perceptions about life in the country of origin and confirms togetherness in the transnational family [23]. Even though actual returns may not match expectations [24], media exposure influences return intentions by spreading events, images, and tales from migrants' home countries. Home countries' provision of dual citizenship to expatriates encourages both remittances and return migration that enable them to better utilize their expatriates' financial and human resources [25]. [26] further noted a combination of drivers and barriers that could determine and shape individuals’ migration scenarios and trajectories, all of which have temporal and spatial effects. However, examination of many studies has shown that little attention is given to the complex nature of factors that can influence the decision to return. Studies by Refs. [[27], [28], [29]] identified a research gap in the factors which influence return decisions. Similarly [24], indicated a major discrepancy between the conceptual and real-life complexities of return migration. The question remains as to how return migration should be integrated into existing frameworks, or whether return migration should be treated as an individual phenomenon. Although research activity about return migration is increasing, there are still research gaps about the factors which influence return decisions in Sub- Saharan Africa. To address this gap, this study examines return migration, specifically drivers and barriers for return migration to Sub- Saharan Africa, based on the evidence from qualitative and quantitative studies. Exploring the barriers and factors of return migration to SSA countries is important because examination of many studies has shown that little attention is given to the complex nature of factors that can influence the decision of return migration. Motivational factors influencing return migration are presented in the review based on the overarching question: why do migrants return to their homeland? Two subsequent research questions are designed to guide the review: 1) what is the driving factor that motivates return migration to sub-Saharan Africa? 2) What barriers restrain migrants from returning to their country of origin? Through a regional literature review, the paper offers the opportunity to contribute to our understanding of the drivers of and barriers to return migration and discuss their embeddedness within migration patterns in the region.

## 2 Theoretical considerations

Return migration encompasses various forms of moving back to one's country of origin, such as repatriation, removal, deportation, assisted return, or even a voluntary return initiated by an individual [24] that can be categorized as either forced or voluntary but can occur anywhere in between. Studies in this field cover a wide range of topics including readmission policy, deportation, theories of return, experiences of return (with a focus on gender), reintegration, and development [24,30,31].

Studies in recent decades have stressed that return migration can be attributed to four primary reasons: 1) inability to assimilate into the host country, 2) personal preference for the home country, 3) attainment of a savings goal, or 4) availability of job opportunities in the home country due to experience gained abroad [[32], [33], [34]]. Additionally, migrants may alter their objectives over time and in response to immigration policies in the host country [35]. While the major determinants of return migration have thus been discussed and described in the literature, it is acknowledged that it is a complex, multidimensional and context-specific process that can have significant impacts on individuals, families, communities, and societies, as well as on the regions and countries involved [36]. Return migration can have impacts on the receiving and sending regions in terms of economic, social, and cultural factors by affecting labour markets, remittance flows, social networks, and cultural norms and practices [37]. Understanding the regional dynamics of return migration is therefore important for policymakers and stakeholders who are concerned with issues related to development, migration, and integration [38]. It thus makes sense to question the determinants of return migration from a regional perspective to understand the possible specific constellations. Studying return migration, especially in sub-Saharan Africa, provides insights into the push and pulls factors that drive return migration as well as the circumstances that could either retain or repel migrants. In Ref. [24] four sets of factors shape considerations about return migration: retention factors and push factors in the country of settlement, and pull factors and repulsion factors in the country of origin. Since the 2000s, there has been a renewed interest in migration, including return migration to SSA [39,40]. Many African countries have experienced improved economic growth and political stability, which can lead to an increase in the number of return migrants [31,41,42]. In addition, there has been a growing recognition of the potential benefits of return migration for both the individual returnees and their countries of origin [43]. These benefits include the transfer of skills, knowledge, and financial resources, as well as the potential for social and cultural integration. Analyzing the recent literature on return migration in SSA published after 2000 will provide important insights into the recent determinants of return migration in the region.

## 3 Methodology

**Fig. 1:**
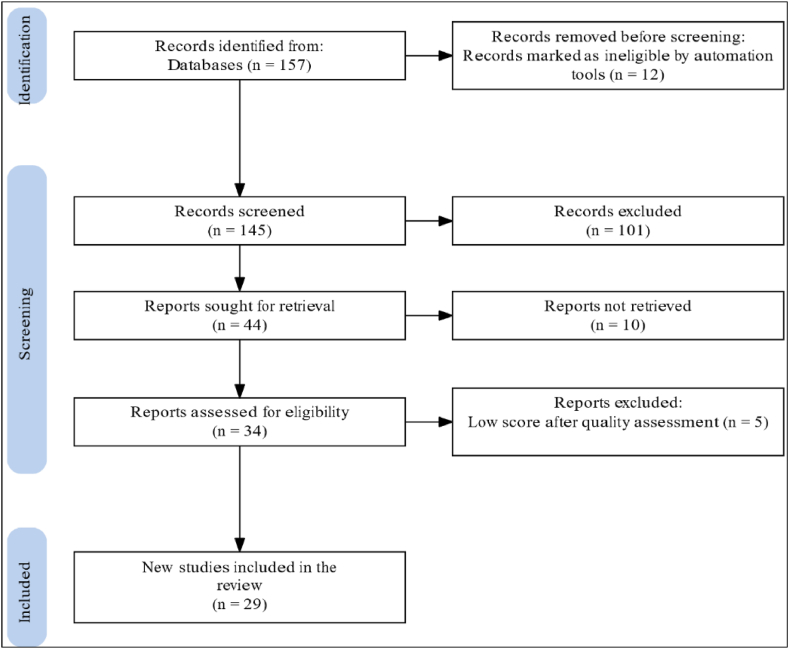
Flowchart showing the systematic approach for the selection of articles reviewed.

**Fig. 2:**
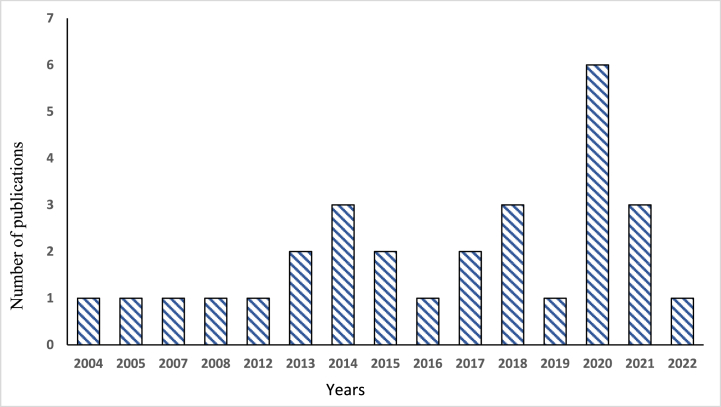
Publications on return migration in sub-Saharan Africa.

A multi-step systematic review of the literature was used in this study. A systematic review approach was used to answer specific research questions for this study, including extensive searching; screening of the articles; synthesizing results; interpreting results, and discussion/reporting findings. The first step of the search was conducted through the following combination of keywords in Scopus and Migration Research Hub, which are the two main databases for the search. Both Scopus and Migration Research Hub were used as search databases because both offer indexed journals, as well as peer-reviewed articles covering the topic of interest. Besides, Migration Research Hub is a widely used hub for issues relating to migration as it provides indexed articles relating to returned migration, leading to its preferred prominence in this field [44,45]. The initial keywords were “return Migration” AND “Sub-Saharan Africa”. Based on the initial keywords, 12 articles were found in Migration Research Hub, and a high number of results were found in Scopus, including unrelated publications from other disciplines and repetitions of those found in Migration Research Hub. To restrict the results, we added further keywords using some common terms relating to return migration and geographical keywords including the names of countries in Sub-Saharan Africa. Thus we use the new keywords – ((“return SAME migra*" OR “forc* AND repatriat*" OR “emigra* OR immigra*" OR “return *Migra*") AND “Africa” AND (“sub-Saharan Africa” OR “Angola” OR “Benin” OR ″ Botswana " OR ″ Burkina Faso " OR ″ Burundi " OR “South Africa” OR “Sudan” OR ″ Sierra Leone” OR “Senegal” OR “Gambia” OR ″ Cote d'Ivoire” OR “Togo” OR “Cameroon” OR “Congo” OR “Niger” OR “Gabon” OR “Zambia” OR “Namibia” OR “Mozambique” OR " Eritrea” OR “Zimbabwe” OR " Djibouti” OR “Rwanda” OR ″ Ghana " OR ″ Cape Verde” OR “Liberia” OR “Guinea” OR “Chad” OR “Somali*" OR “Nigeria” OR “Madagascar” OR " Ethiopia” OR ″ Guinea-Bissau " OR " Kenya” OR “Equatorial Guinea " OR ″ Malawi " OR ″ Mali " OR ″ Mauritania " OR ″ Tanzania " OR ″ Uganda " OR ″ Mauritius " OR ″ Sao Tome and Principe " OR ″ Central African Republic " OR “Seychelles” OR “Eswatini” OR ″ Lesotho ")). This resulted in the most up-to-date list of publications found in Scopus. Thus, the search results yielded 157 studies published from the years 1984 and onwards and included all articles published up to 2022 (Fig. 1). The searching and sampling procedure followed similar phases, as was done in the migration review by Refs. [46,47]. Some papers on return migration were considered with a slight variation of words through hand searching. We observed some duplication of articles (12 articles) that were found in both Scopus and Migration Research Hub, and this was rectified. In the second step, the title and abstracts of the 145 records and the hand search results were screened for duplicates. The authors then chose 44 published articles based on their relevance to the topic. Out of those, 34 papers were left to be assessed based on their theme and significance after 10 studies were eliminated, eight of which were discarded due to topical similarity and two of which were not geographically focused on SSA countries. In the last step, the 34 publications that were identified went through a quality check following an adapted quality-assessment protocol by Ref. [48] on conceptual framing and methodological transparency, which resulted in the elimination of 5 studies with low scores. Hence, a total of 29 studies (see Fig. 1, Fig. 2), were selected for the review, and Mendeley software was used to process the scholarly articles. Only publications that focus on factors, reasons, motives and determinants for international return migration to or within sub-Saharan Africa were included in the sample. Overall, the remaining articles that were assessed were written between 2000 and 2022 as no relevant article was found in the literature before 2000.

Thematic analyses were conducted on the sampled articles. All articles were skim-read and tested for their thematic relevance based on their titles, abstracts and full texts, grounded on the research question and overall aim of the study. Generally, multiple coders were used to gain a broad range of factors of return migration to sub-Saharan Africa and to analyse the similarities observed among the different studies. The authors coded articles separately at the initial stage and later came together, thus articles' contents were thematically coded for both qualitative and quantitative (Qual-Quant) studies and those studies that used mixed methods. These analytical methods led to the development of distinctive discussion structures (structural factors, individual & social factors, and policy factors) that were recognized from the thematic areas that emerged from analysing the articles’ content. The observed factors to return migration to SSA were analysed and discussed based on categories of structural, individual and policy issues as classified by Ref. [32].

## 4 Results and discussion

### 4.1 Geographical scope, data and methods of the selected studies

**Fig. 3:**
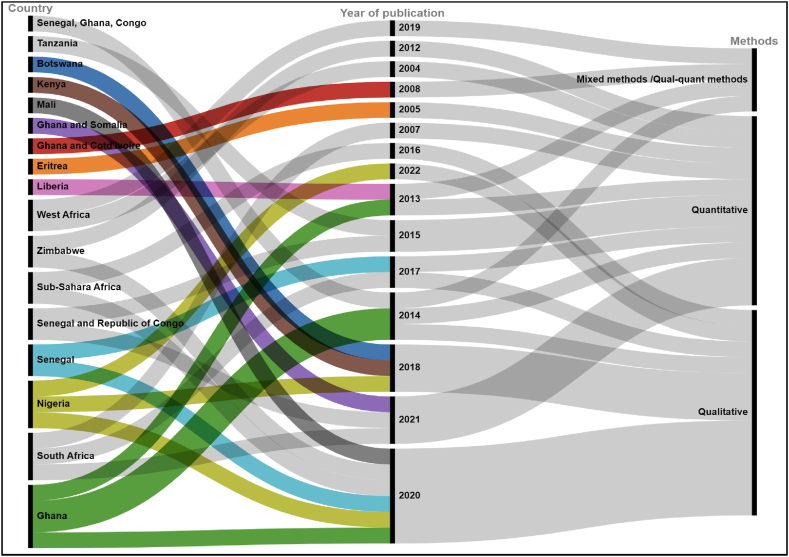
Spatio-temporal and methods used in the selected studies.

The results show that the geographical locations of countries of destination are almost all countries in the European Union (particularly those with colonial and language affiliations), the United Kingdom, China, and within Africa, the major destination being South Africa. As migration decisions are triggered by various factors, the return decisions of migrants are also determined by numerous complex drivers and barriers. The number of publications related to return migration has increased over the past two decades. In the sample, the first paper was published in 2004, and an increasing trend in publications is noticeable from 2013 onwards. The increase becomes even more significant from 2014 onwards, reaching a peak in publications in 2020 (see Fig. 1). In terms of methodological approach, most studies used either qualitative or quantitative methods, but some used mixed-methods approaches. Out of the total selected studies, 12 of them [[49], [50], [51], [52], [53], [54], [55], [56], [57], [58], [59], [60]] were used quantitative approach. Whereas 13 studies were qualitative [[11], [12], [13],^49^,[61], [62], [63], [64], [65], [66], [67], [68]]. A mixed-methods approach was also used in 4 studies [[69], [70], [71], [72]] (see Fig. 3).

The review of the studies mainly addressed Sub-Saharan countries. Four studies focused exclusively on return migration to Ghana [50,61,62,69] whereas [73] address how African immigrants in Canada, mainly from Ghana and Somalia, contemplate the decision to return to their homelands, and [70] addressed Ghana and Cote d’Ivoire. Three studies [52,53,68] emphasized return migration to South Africa. Senegal and other SSA nations are covered in five studies. Two studies [60,65] address how the aspiration for religious education and migration-policy changes in Europe influence the return decisions of Senegalese migrants. Three studies engaged with return-migration decisions and issues related to the reintegration problem upon return in multiple countries including Senegal and the Republic of Congo [57,58] as well as [74] were covered Senegal, the Republic of Congo and Ghana. Two studies in the case of Zimbabwe [54] and three studies in Nigeria [66,67,75] investigated the perceptions and intentions to return, while [71] discovered the complexity of return decisions in case of Liberia. While one study each covered Botswana [13], Mali [63], Eritrea [59], and Tanzania [55]. However, a case study from Kenya explored the centrality of identity in return migration [49]. Exceptionally, certain studies (see Fig. 3) address a wide range of geographical scope in sub-Saharan [11,64] and in West Africa [13,56].

### 4.2 Evidence on drivers of and barriers to return migration to sub-Saharan Africa

Based on the theoretical and empirical reviews, intentions that shape the decision to return to sub-Saharan Africa are frequently influenced by a broad range of factors both from the origin and destination countries. These include personal, sociocultural, political and economic circumstances as well as policy interventions. As summarized by Ref. [32] in a framework, the range of factors which shape return decisions is classified in Table 1. Hence, the factors are summarized and discussed in the following sections 1) structural factors, 2) personal and social factors, and 3) policy interferences. Generally, from Tables 1 and it is obvious that individual and social factors, mainly family reasons, are the prominent driving factors of return migration to SSA. Among structural factors, unemployment-related causes are highly influential drivers, followed by the achievement of an economic goal that consists of good savings and positive migration outcomes. Immigration policies associated aspects are noted in the studies to be influential among the policy interference factors, with restrictive immigration policies among the major barriers that limit the possibilities of remigration. The political and socio-economic situation at home also plays a key role in return decisions (Table 1).

**Table 1:** Overview of drivers & barriers to return to sub-Saharan Africa.

Dynamics of return migration	Category	Factors to return	Frequency
Drivers	Structural factors	Unemployment	11
		Achieving economic goals/savings, positive migration outcome	10
		Economic opportunity at the origin	9
		Integration difficulties at the destination	5
		Investment	3
		The political and economic problems at the destination	3
		Education opportunity at the origin	2
		Retirement	2
		Political stability at the origin	1
		Liberation	1
	Individual and social factors	Family reasons	24
		Homesickness, emotional links to homeland	6
		Inculcate culture, religion & communal life	5
		Personal aspirations	4
		Racism, exclusion, and prejudice	4
		Educational reasons	4
		Social network lead opportunity	2
		Serious illness	2
		Perceive quality life in the homeland	1
		Failure migration experience	1
		Negative decisions about asylum	1
	Policy factors	Destination countries' migration policies	6
		Lack of legal status	3
		Presence of legal status/possibility of remigration	1
Barriers	Structural factors	The political and socio-economic situation at home	5
		Economic success & better working conditions at the destination	3
		Unemployment and unfavourable circumstances in the origin	2
		Economic failure at the destination	1
	Individual and social factors	Unwilling to return empty-handed back home	3
		Personal reasons and feelings of exclusion	3
		Family reasons	4
		A good living and education for children at the destination	2
		Strong diaspora family ties	1
		Social freedoms at the destination	1
		Restrictions of mobility freedoms at the origin	1
	Policy factors	Restrictive immigration policies/limit the possibility of remigration	6
		Legal rights in destination	1

#### 4.2.1 Structural factors

Decisions regarding return migration could be determined by numerous structural factors, such as economic, political, social as well as environmental circumstances both at the place of origin and at the destination. In sub-Saharan Africa, factors associated with economic issues and political situations were found to play a vital role in people's migration histories [8,12,26,41,63,[76], [77], [78], [79], [80]]. Economic, political, and environmental crises in countries of origin are among the principal causes of unfavourable living conditions that prompt people to migrate elsewhere [53,54,60,74]. Differences in employment opportunities and wage rates are among the potential economic factors people consider when deciding either to migrate or return [70]. In SSA, the return decision of migrants is influenced both by economic failure and success (Table 1). It is likely that migrants will consider return migration if they either achieve or fail to achieve their economic goals in their places of destination, or if better economic opportunities and success are expected in their places of origin [70,81]. The successful acquisition of enough financial and human resources is found to be a motive for return in numerous studies [13,50,57,58,60,61,69]. The desire to invest their savings actively in the local economy in the domestic sphere could be a motivational attraction from the country of origin that drives return [50,57,62]. For instance, economic attainment at the place of origin has been stated as the trigger factor for return in Ghana [69].

[70] for example found that employment experience overseas is the most important predictor of investment/entrepreneurial activity among return migrants polled. On the other hand, the findings by Ref. [50] in Ghana and by Ref. [56] in other West African countries revealed that a desire to invest in the country and family ties were important pull factors in the decision to return. However, poor job-market outcomes [55,56,81], unemployment, and difficulties with integration at the destination increase the likelihood of the intention to return [50,60]. In contrast, migrants who are successfully integrated economically into the destination may reverse return decisions [68]as better income and good working and living situations are among the primary reasons to stay [13]. Issues of employment, political unrest, an unstable economy, and potential difficulties in reintegrating into society in the homeland made emigrants reluctant to return [12].

On the other hand, unsatisfying work circumstances and unemployment in places of destination can initiate the home return. However, failure to achieve economic success due to unemployment, high cost of living, or low income prolonged stay in the destination [50,54,60]. Furthermore, the possibility of returning home empty-handed is a barrier to return, as doing so would constitute personal failure and loss of pride, resulting from high expectations from members of the family [62] that migrants meet the expectations of the “success social construct” [68]. Likewise, failing to meet the “success social construct” due to the inability to overcome adversity in the host country [68] blocked Zimbabwean migrants from returning. The comparative advantage of the low cost of living at the place of origin over costly living abroad, and also the possibility of retirement, also inspire the decision to return home [82,83]. Liberation coupled with family issues and political situations at the destination were found to be the main driving forces [59] for Eritrean and Senegalese returnees [60]. As specified by Ref. [63] economic and security problems at their destinations triggered the return decisions of Malian migrants, while Liberian migrants' reluctance to return was caused by the unstable political and economic situation in their country of origin [71]. The presence of exclusion and prejudice [62], and issues related to cultural and identity clashes including differences in food type and feeding habits, as well as language barriers [49,61] in countries of destination force migrants to return. Duration of stay and distance influence return decisions; the likelihood of return becomes lower as the distance and duration of stay become longer [54,74]. In sub-Saharan Africa, various structural factors such as conflict, violence, and economic crisis, as well as volatile political situations at the place of origin are found to be potential barriers to return [11,12,50].

#### 4.2.2 Personal and social factors in return migration

Individual and social factors include social ties, homesickness, family and responsibility for children and relatives, which are primarily influencing return decisions [13,50,54,[57], [58], [59], [60],^64^,^66^,^67^,^69^,^74^]. Personal and family matters [56,59,68] such as making a family, family unity, the need for taking care of sick parents, accommodating siblings after the death of parents, and the desire to connect and live with ageing parents or members left behind at home inspire migrants to return [61,66,67,69,81,84]. Family breakup is reported to prompt the return decisions of women returnees in one case study [55]. Some migrants prefer to stay in their place of destination [62] due to the favourable environment for bringing up their children, whereas others want to return, believing that the upbringing of their children will be easier and better in their homeland in their own culture and religion [11,62,65]. For example, access to religious education was found to be the main reason that Senegalese migrants sent their children back home from New York [65].

The evidence found regarding educational opportunities for children and return decisions is contradictory, which could be the result of different individual preferences and beliefs. That could imply that the issue of education as a factor influencing the decision to return may be both/a barrier and/or a driving factor. The evidence further implied the dual role that family played in return migration, as a pull factor from the origin, and as a barrier when family are present in the place of destination. The presence of family at the destination was a reason to stay [68] whereas the desire to form a family, unification with family and emotional links to families back home are motivations influencing the decision to return [13,59,64]. While [67] discovered family issues to be both an attraction for individuals who left their families behind and a barrier to returning for those who established families at the destination. The individualistic way of living in the modernized parts of the globe on the one hand, and the demand for a communalistic life and social well-being in the country of origin on the other, drives Black Africans to return [64]. Case study results from Botswana migrants [13] show that homesickness and family primarily initiate their return decisions. Serious illness is reported to drive the decision to return home where strong social networks and support systems exist, to either recover or, perhaps, die [52]. A family crisis like the death of a family member is another circumstance that may be a reason for returning permanently [13,75]. Another factor that influences migrants’ decisions to return is the state of employment. Unemployment was found to be a driving factor to return home for migrants in various countries, including migrants from Senegal [60], South Africa [54] and Ghana [50]. For instance, in rural Tanzania return is linked to poor job-market outcomes at the destination [55].

A combination of individual and social attributes is found to influence both drivers of and barriers to return, whereas the return-decision process is found to be more influenced by social factors in the majority of the studies in the review. The social factors include family ties, family formation, and unification; the desire to live with ageing people; taking care of parents, siblings, and children left behind; homesickness; the need for communalistic life, identity, and culture; and a strong social support system in the domestic place; all are found to be major reasons for returning [50,52,59,60,71]. However, contradictory evidence has been found concerning family reasons [56,74]. reported that family in the destination do not influence return decisions, whereas [57] collected empirical evidence to the contrary.

#### 4.2.3 Policy interferences

Restrictive immigration policies influence migration and return decisions [53,60,74] as border control and exit policies have become more restrictive [85]. Migration policies in origin and destination countries affect migration, and restrictive immigration policies tend to delay and reduce return [74]. Some studies show that a considerable number of migrants use irregular routes and transits because of limited migration possibilities, including the demand for formal documents [[86], [87], [88]]. Within the thematic discussions of the articles reviewed, there are differences in policy characteristics in both countries of destination and origin, as this affects the return-migration decisions. The different characteristics of destination countries' policies influence on return migration to SSA have played a role on many occasions, as reported in the return migration literature [38,53,70,89]. Migrants’ legal status at the place of destination influences the return decision. For example, being unable to integrate into the legal system, and the absence of permissible status in the destination drives migrants to return to their domestic places [49,53]. Government restrictions at the destination have been reported as the main reason for return by Ref. [50].

Moreover, some of the authors note that distance and duration influence the return decision: the greater the distance and the longer the duration at the destination, the lower the likelihood of return [53,74]. A long migration trajectory sometimes also entails higher risks and greater financial expenses; these factors may negatively influence the return decision because the high personal and financial costs of migration must be transformed into value. Other reasons which are potentially related to the political and socio-economic situation of a country and hinder a return to sub-Saharan Africa are “institutional crisis”, “war and conflict”, “criminality”, and bad “working conditions” [11] and the “fear of traffickers” due to debts and reciprocity [75]. Also, cultural aspects are mentioned as additional driving forces for return, such as food and language [61]. Some studies also revealed cultural “clashes” between Western and sub-Saharan values and cultural habits [49], “exclusion and prejudice” [62] or expensive childcare [13] as other reasons for return.

Overall, individual factors, especially social ones, seemed to be most vital in the return-decision process, which pointed to a major difference in the drivers underlying migration decisions. Furthermore, despite the growing interest in environmental drivers of migration [14,90], it is interesting to note that none of the studies explicitly mentions environmental or climate drivers as determinants in the context of return migration. Yet we might imagine that environmental and climate risks can become significant determinants of return migration. Climate change and environmental hazards such as natural disasters, droughts, and floods can adversely affect the livelihoods of migrants in their host countries [91], especially if they are working in vulnerable sectors such as agriculture or informal settlements. Such risks can exacerbate existing economic and social vulnerabilities, making it difficult for migrants to sustain themselves and their families in their host countries [92]. As a result, some migrants may choose to return to their countries of origin, where they may have a better understanding of the local environmental and climatic conditions and may be better equipped to cope with the risks.

## 5 Conclusion

The literature review, based on 29 studies dealing with return migration to SSA published between 2000 and 2022, has provided a detailed overview of the various drivers that initiate, support, hinder or delay return migration in the region. Structural, social, individual, and policy factors from both sides – the destination countries and the countries of origin – shape the decision to return to SSA. Opportunities, living conditions, and the political and economic situations of the destination country as well as the country of origin are scrutinized before a decision is made. In line with the literature on the subject [24,56,70] and the determinants highlighted in other regions of the world [93], the analysis pointed out that the return decision is not only based on rational factors but is often also shaped by emotional and social factors such as longing for family or homesickness. The evidence of our regional review, therefore, suggests that return migration seems to be more socially and emotionally driven, contrary to the (out) migration process, which is primarily led by economic reasons. Some factors – such as economic and political instabilities, crises and conflicts – which may be at the origin of the decision to migrate continue to be a reason for migrants not to return to their place of origin. Yet social and personal factors including family ties, social identity, and the desire to regain one's home culture have a crucial influence on the return decisions, sometimes supplanting economic drivers. Prior migration experience and the risks taken to migrate are also considered when migrants plan to return.

What is apparent from the present study is that, though numerous studies have been conducted over the past decades, they only constitute a limited representation, as they mainly focus on a specific country, a specific group of people, and a certain aspect of return migration. This structural lack of data might be due to the difficulties in recruiting participants, as it is a great challenge to reach return migrants, and it seems to be nearly impossible to establish a balanced set of participants [74]. Thus, to contribute to the knowledge gap about return migration, in the present study, we reviewed and précised the findings of studies about return migration to sub-Saharan Africa. This geographical focus was chosen based on the following considerations: first, migration within and from this region has risen tremendously over the last two decades; in 2019, more than 21 million sub-Saharan African migrants were living in another African state, and about 19 million were living on another continent [3]. This implies that migratory trajectories originating from this region may have considerable effects on countries of destination and origin. Return migration back to the region may be considerable, and therefore worth investigating. Unfortunately, no precise numbers for return migration to Sub-Saharan Africa exist in the literature. Another argument for this decision was that other researchers have already used the same geographical scope for their studies, for example [11] investigate migration between sub-Saharan Africa and Europe.

Finally, the study underscores the importance of continued research on return migration to deepen our understanding of this critical aspect of global migration. An important point for regional stakeholders is that policy incentives and disincentives, such as information about return possibilities and support schemes, seem to play a minor role in shaping return migration, while general migration restrictions constitute a barrier for some potential returnees.

## 6 Limitations

One of the limitations of this study is that the results presented here were highly dependent on the methodological design of the study, as the authors selected only articles indexed by Scopus and Migration Research Hub only.

## Declarations

### Author contribution statement

All authors listed have significantly contributed to the development and the writing of this article.

### Data availability statement

Data will be made available on request.

### Additional information

Correspondence and requests for materials should be addressed to Lemlem F. Weldemariam.

## Declaration of competing interest

The authors declare no competing interests.

## Figures and Tables

**Table:** 

Principles	Questions	Scoring
Conceptual framing	a) Does the study have a conceptual framework and clear research question? b) Does the study appear to draw conclusions based on its results rather than theory or policy?	0 Neither 1 One 2 Both
Methodological transparency	a) Does the study explain its research design and data collection methods? b) Does the study present or link to data sources?	0 Neither 1 One 2 Both
Internal and external validity	a) Is the study internally valid? Or, are alternative causes of impact or the study's limitations considered? b) Is the study externally valid? Or, can findings be generalised to other contexts and populations?	0 Neither 1 One 2 Both
Journalistic	a) How relevant is the study to the research topic?	1 Partially 2 Directly
Score (Sum)		0–8

Scoring: 0–4 Low [Excluded] 5–6 Medium 6–8 High.

## Appendix

Adapted quality-assessment protocol applied for this review [48]
